# Acute Illness Associated with Use of Pest Strips — Seven U.S. States and Canada, 2000–2013

**Published:** 2014-01-17

**Authors:** Rebecca J. Tsai, Jennifer Sievert, Joanne Prado, Incident Reporting Program, Kaci Buhl, Dave L. Stone, Mathias Forrester, Shelia Higgins, Yvette Mitchell, Abby Schwartz, Geoffrey M. Calvert

**Affiliations:** 1 Div of Surveillance, Hazard Evaluations, and Field Studies, National Institute for Occupational Safety and Health, CDC; 2 Washington State Dept of Health; 3 Pest Management Regulatory Agency, Health Canada; 4 National Pesticide Information Center; 5 Texas Dept of State Health Svcs; 6 North Carolina Div of Public Health; 7 New York State Dept of Health; 8 Michigan Dept of Community Health

Dichlorvos-impregnated resin strips (DDVP pest strips) are among the few organophosphate products still available for indoor residential use. The residential uses for most other organophosphate products, including most DDVP products, were canceled because they posed unreasonable risks to children (1). DDVP pest strips act by inhibiting acetylcholinesterase activity in the brain and nerves of insect pests and are designed to gradually release DDVP vapor for up to 4 months (2,3). Acute illnesses in humans associated with nonlethal acute exposures usually resolve completely, but recovery is not always rapid (2). To assess the frequency of acute illnesses associated with DDVP pest strips, cases from 2000 through June 2013 were sought from the 12 states that participate in the Sentinel Event Notification System for Occupational Risks (SENSOR)–Pesticides Program, the National Pesticide Information Center (NPIC), and Health Canada.* A total of 31 acute DDVP pest strip–related illness cases were identified in seven U.S. states and Canada. The majority of these illnesses resulted from use of the product in commonly occupied living areas (e.g., kitchens and bedrooms), in violation of label directions. Although 26 of the 31 cases involved mild health effects of short duration, five persons had moderate health effects. Illnesses caused by excess exposure to DDVP pest strips can be reduced by educating the public about the proper usage of DDVP pest strips and with improvements in label directions.

Cases were defined and classified based on the strength of evidence for DDVP exposure and health effects consistent with and following exposure to DDVP pest strips.† Information was collected on demographic characteristics, event location, health effects, outcomes (e.g., hospitalization), contributing factors, reporting source, illness severity,§ and work-relatedness.

From 2000 to 2013, a total of 31 (30 possible and one probable) cases of acute DDVP pest strip–related illness were identified in the United States (24 cases) and Canada (seven). The 24 U.S. cases were reported to SENSOR or NPIC from seven states; the seven Canada cases were reported to Health Canada from across the country. Twenty-six (84%) of the 31 cases were classified as of low severity, and 24 (77%) of the patients were female (Table). Among the 22 cases for which age was known, the mean age of patients was 48 years. Twenty-four (77%) of the exposures occurred in private residences. The most commonly reported affected body systems and their symptoms were neurologic (68%) (e.g., headache), respiratory (55%) (e.g., dyspnea), and gastrointestinal (42%) (e.g., nausea) (Table). Five of the 31 persons had health effects considered moderate, including asthma attack, respiratory distress requiring hospitalization, paresthesias, and incoordination.

A total of 20 (65%) of the 31 cases involved label¶ violations, mostly use of DDVP pest strips in areas occupied by persons ≥4 hours/day. For the remaining 11 cases, information was not sufficient to determine if whether usage of DDVP pest strips resulted in a label violation (Table). Contributing factors other than using strips in occupied areas included excessive application (two cases), placing strips in sealed bags to treat infested items (four), lack of skin protection (e.g., gloves or prompt skin washing) (four), placing strips in closets and pantries (three), cutting and tearing strips into smaller pieces (three), and using a heater and fan to accelerate vapor dissemination from strips (three).

In the 11 cases for which it was unclear whether a label violation had occurred, exposure might have resulted from misunderstanding of label directions. Currently in the United States, DDVP pest strips are offered in three different sizes: 16 g, 65 g, and 80 g. Label directions differ across sizes, but also can differ across brands of the same size. For example, whereas all labels specify that one 65 g or 80 g strip will treat up to 900–1,200 cubic feet, not all labels advise against using the product in smaller spaces, nor do they all provide a clear warning against excessive application. Moreover, some labels list offices as appropriate places for strip placement even though these are typically occupied for ≥4 hours/day. Finally, although some strips are approved for bed bug control, the directions for use are substantially different for bed bugs versus other insect infestations, which might confuse some users and lead to improper use.** Preventing DDVP pest strip–related illnesses requires educating the public regarding how to correctly use DDVP pest strips and how to control insect pests using methods with the least possible health and environmental hazards.

## Figures and Tables

**TABLE:** Characteristics of patients (N = 31) with acute dichlorvos (DDVP) pest strip–related illness — seven U.S. states and Canada, 2000–2013

Characteristic	No.	(%)
**Age group (yrs)**		
≤19	1	(3)
20–64	24	(77)
≥65	4	(13)
Unknown	2	(6)
**Sex**		
Female	24	(77)
Male	7	(23)
**Body system/Organ affected** *		
Neurologic	21	(68)
Respiratory	17	(55)
Gastrointestinal	13	(42)
Other	11	(35)
Skin	7	(23)
Eye	7	(23)
**Case classification**		
Possible	30	(97)
Probable	1	(3)
**Severity of illness** †		
Low	26	(84)
Moderate	5	(16)
**Route of exposure** §		
Respiratory	28	(90)
Dermal	5	(16)
**Location of exposure**		
Home	24	(77)
Workplace (Store/Office)	3	(10)
Other (Boat/Car)	3	(10)
Unknown	1	(3)
**Label violation status**		
Applied DDVP in areas occupied by humans ≥4 hours/day	18	(58)
Excessive application	2	(6)
Undetermined¶	11	(35)

* Sum exceeds 100% because some patients had more than one affected body system/organ.

† Low severity cases usually resolve without treatment and cause minimal time lost from work (<3 days). Moderate severity cases are not life threatening but require medical treatment and result in <6 days lost from work.

§ The sum exceeds 100% because two cases had both routes of exposure.

¶ Insufficient data were available to determine whether the DDVP strip usage resulted in a label violation.

## Footnotes

* SENSOR-Pesticides consists of state health departments in 12 states that conduct surveillance of pesticide-related illness (http://www.cdc.gov/niosh/topics/pesticides/overview.html). Five of the 12 states reported acute DDVP pest strip–related illness (Michigan, North Carolina, New York, Texas, and Washington); the other seven states (California, Florida, Iowa, Louisiana, Nebraska, New Mexico, and Oregon) did not identify any acute DDVP pest strip–related illness during 2000–2013. NPIC, an organization that provides pesticide-related information to the public and health-care professionals and captures human/animal pesticide exposure incidents reported by callers, reported acute DDVP pest strip–related illness from four states (New Mexico, New York, Ohio, and Texas)(http://npic.orst.edu/about.html). There was no overlap in the New York and Texas cases reported by SENSOR-Pesticides and NPIC.

† Probable cases are based on a mix of objective and subjective data about exposure and health effects, and possible cases are based on subjective exposure and health effects data. The complete case definition is available at http://www.cdc.gov/niosh/topics/pesticides/pdfs/casedef.pdf.

§ Standardized coding was used to determine severity of illness (available at http://www.cdc.gov/niosh/topics/pesticides/pdfs/pest-sevindexv6.pdf). Low severity cases usually resolve without treatment and cause minimal time lost from work (<3 days). Moderate severity cases are not life threatening but require medical treatment and result in <6 days lost from work.

¶ Pesticide product labels provide legally enforceable information on how to safely handle and apply pesticides. The label is the law. The U.S. Environmental Protection Agency approves the labels and enforces them with the assistance of state agencies.

** Control of bedbugs involves placing the bed bug–infested items in a sealed bag along with the strip, whereas for control of other insects, the strip should be hung in the desired location.
